# 

**Published:** 1905-02

**Authors:** 


					﻿ATLANTA
JOURNAL-RECORD
ra s Qp MEDICINE.
SUBSCRIPTION SI.OO,
PUBLISHED MONTHLY
Successor to Atlanta Medical and Surgical Journal, Established 1855,
and Southern Medical Record, Established 1870.
Vol. VI.
Atlanta, Ga., February, 1905.
No. 11’.
				

